# The Environmental Stability of SARS-CoV-2 Variants Omicron BA.1 and BA.5 on the Surfaces of Widely Used Transport Packaging Materials

**DOI:** 10.1128/spectrum.04881-22

**Published:** 2023-04-10

**Authors:** Bei Wang, Lan Chen, Hongtao Sui, Xiaojing Dong, He Huang, Xinming Wang, Yan Xiao, Xia Xiao, Chao Wu, Hong Gao, Yuelong Shu, Lili Ren, Jianwei Wang

**Affiliations:** a National Health Commission Key Laboratory of Systems Biology of Pathogens and Christophe Mérieux Laboratory, Institute of Pathogen Biology, Chinese Academy of Medical Sciences & Peking Union Medical College, Beijing, China; b National Health Commission Key Laboratory of Systems Biology of Pathogens, Institute of Pathogen Biology, Chinese Academy of Medical Sciences & Peking Union Medical College, Beijing, China; c Key Laboratory of Human Disease Comparative Medicine, Chinese Ministry of Health, Beijing Key Laboratory for Animal Models of Emerging and Remerging Infectious Diseases, Institute of Laboratory Animal Science, Chinese Academy of Medical Sciences and Comparative Medicine Center, Peking Union Medical College, Beijing, China; d Key Laboratory of Respiratory Disease Pathogenomics, Chinese Academy of Medical Sciences and Peking Union Medical College, Beijing, People’s Republic of China; Shandong First Medical University

**Keywords:** SARS-CoV-2, Omicron variants, environmental stability, transmission, surface

## Abstract

The increased transmissibility of SARS-CoV-2 variants of concern (VOCs) has raised questions regarding the environmental stability of these viruses. Although a prolonged survival time has been reported for SARS-CoV-2, how long new variants can persist on contaminated surfaces and how environmental factors affect the persistence time are not fully characterized. The present study provides a comprehensive assessment of the stability of Omicron variants BA.1 and BA.5, which are currently circulating strains, on the surfaces of widely used transport packaging materials. By monitoring viable virus detection over a 7-day period under different environmental conditions, it was found that the environmental stability of SARS-CoV-2 Omicron variants depended heavily on the surface type, temperature, and virus concentration. In addition, virus nucleic acid exhibited high stability on the material surface independent of whether viable virus was detected. These findings provide useful information for logistics practitioners and the general public to appropriately deal with transport items under different conditions to minimize the risk of epidemic transmission.

**IMPORTANCE** This study shows the environmental stability of SARS-CoV-2 Variants Omicron BA.1 and BA.5 on surfaces of widely used transport packaging materials. The findings demonstrate that the environmental stability of the SARS-CoV-2 Omicron variants varies based on material type. The viability of SARS-CoV-2 on material surfaces depends heavily on temperature and viral titer. Low temperatures and high viral titers promote virus survival. Moreover, in contrast to virus viability, virus nucleic acid exhibits high stability on the surfaces of widely used materials, making the detection of virus nucleic acid unsuitable for evaluating the risk of epidemic transmission.

## INTRODUCTION

Since the first emergence of the SARS-CoV-2 virus in November 2019, various variants of concern (VOC) have emerged and spread globally. Among these VOCs, the currently circulating Omicron strain is a major public health concern owing to its high infectivity and transmissibility (1). The increased infectivity and transmissibility of SARS-CoV-2 VOCs can be explained by several factors, such as increased host receptor binding affinity and prolonged viral shedding from infected individuals. Increased environmental stability is also a key point of concern (2). SARS-CoV-2 VOCs with increased environmental persistence are more likely to be transferred and cause onward contact transmissions. Trade and travel customs and the express and logistics industry face great challenges due to this possibility. Appropriate measures should be taken to minimize the risk of contact infection caused by express delivery and by imported and exported cargos.

In this study, the stability of two Omicron subvariants, BA.1 and BA.5, on the surfaces of four widely used transport packaging materials were comprehensively evaluated over a 7-day period. Other variables, including the viral concentration, temperature, and relative humidity (RH), were also included to precisely assess situations under different conditions. An understanding of the environmental stability and persistence of SARS-CoV-2 Omicron variants on contaminated surfaces has profound impacts on the handling of transport items.

## RESULTS

The virus viability of Omicron subvariants on the surfaces of four widely used transport packaging materials was investigated over time. There were three temperature conditions in this study: 4°C (low temperature), 25°C (room temperature), and 37°C (high temperature). At each temperature, 180 samples were recovered for each subvariant, including four material surfaces, five time points, and three RH conditions. At 4°C, 47 BA.1 samples and 68 BA.5 samples were detected with the viable virus (Table 1); at 25°C, the numbers of positive samples of BA.1 and BA.5 were 20 and 29, respectively (Table 2); and at 37°C, the numbers of positive samples decreased to four BA.1 samples and five BA.5 samples (Table 3). These results indicated that the virus was highly stable at the low temperature, and that the viable virus could be recovered from the surfaces of nonwoven fabric, polyethylene (PE) packaging film, and iron even on day 7. In addition, considering the fact that more samples were detected with viable viruses from samples using BA.5 (102, 56.7%) than BA.1 (71, 39.4%) (Table 1 to 3), the results indicated that the environmental stability of BA.5 was slightly greater than that of BA. 1.

**TABLE 1:** Viable virus detected at 4°C^*a*^

			Virus titer														
			1 (Log_10_TCID_50_)					2 (Log_10_TCID_50_)					3 (Log_10_TCID_50_)				
Temp	Materials	RH^*b*^	6h	1d	3d	5d	7d	6h	1d	3d	5d	7d	6h	1d	3d	5d	7d
4°C	Paper carton	40%	−/−	−/−	−/−	−/−	−/−	+/+	−/−	−/−	−/−	−/−	+/+	+/+	−/−	−/−	−/−
		60%	−/−	−/−	−/−	−/−	−/−	−/+	−/−	−/−	−/−	−/−	+/+	+/+	−/−	−/−	−/−
		80%	−/−	−/−	−/−	−/−	−/−	−/−	−/−	−/−	−/−	−/−	+/+	+/+	−/−	−/−	−/−
	Nonwoven fabric	40%	−/−	−/−	−/−	−/−	−/−	+/+	+/+	−/−	−/−	−/−	+/+	+/+	+/+	+/+	−/+
		60%	−/−	−/−	−/−	−/−	−/−	+/+	−/+	−/−	−/−	−/−	+/+	+/+	+/+	+/+	−/+
		80%	−/+	−/−	−/−	−/−	−/−	+/+	+/+	−/−	−/−	−/−	+/+	+/+	+/+	+/+	−/−
	PE packaging film	40%	−/+	−/−	−/−	−/−	−/−	−/+	−/+	−/−	−/−	−/−	+/+	+/+	+/+	+/+	−/+
		60%	−/+	−/−	−/−	−/−	−/−	−/+	−/+	−/−	−/−	−/−	+/+	+/+	+/+	+/+	−/+
		80%	−/−	−/−	−/−	−/−	−/−	+/+	+/+	−/−	−/−	−/−	+/+	+/+	+/+	−/+	−/−
	Iron	40%	−/−	−/−	−/−	−/−	−/−	−/+	−/+	−/−	−/−	−/−	+/+	+/+	−/+	−/+	−/+
		60%	−/−	−/−	−/−	−/−	−/−	+/+	−/+	−/−	−/−	−/−	+/+	+/+	+/+	+/+	−/+
		80%	−/−	−/−	−/−	−/−	−/−	−/−	−/−	−/−	−/−	−/−	+/+	+/+	+/+	−/−	−/−

a −/− stands for no viable virus detected for both BA.1 and BA.5; −/+ stands for viable virus detected for BA.5 but not BA.1; +/+ stands for viable virus detected for both BA.1 and BA.5.

b RH, relative humidity.

**TABLE 2:** Viable virus detected at 25°C^*a*^

Temp	Materials	RH^*b*^	Virus titer														
			1 (Log_10_TCID_50_)					2 (Log_10_TCID_50_)					3 (Log_10_TCID_50_)				
			6h	1d	3d	5d	7d	6h	1d	3d	5d	7d	6h	1d	3d	5d	7d
25°C	Paper carton	40%	−/−	−/−	−/−	−/−	−/−	−/−	−/−	−/−	−/−	−/−	−/+	−/−	−/−	−/−	−/−
		60%	−/−	−/−	−/−	−/−	−/−	−/−	−/−	−/−	−/−	−/−	+/+	−/+	−/−	−/−	−/−
		80%	−/−	−/−	−/−	−/−	−/−	−/−	−/−	−/−	−/−	−/−	−/+	−/−	−/−	−/−	−/−
	Nonwoven fabric	40%	−/−	−/−	−/−	−/−	−/−	−/+	−/−	−/−	−/−	−/−	+/+	+/+	−/−	−/−	−/−
		60%	−/−	−/−	−/−	−/−	−/−	−/+	−/+	−/−	−/−	−/−	+/+	+/+	−/−	−/−	−/−
		80%	−/+	−/−	−/−	−/−	−/−	+/−	−/−	−/−	−/−	−/−	+/+	+/+	−/+	−/−	−/−
	PE packaging film	40%	−/−	−/−	−/−	−/−	−/−	−/−	−/−	−/−	−/−	−/−	+/+	+/+	−/−	−/−	−/−
		60%	−/−	−/−	−/−	−/−	−/−	+/−	−/−	−/−	−/−	−/−	+/+	+/+	−/−	−/−	−/−
		80%	−/+	−/−	−/−	−/−	−/−	−/−	−/−	−/−	−/−	−/−	+/+	+/+	−/+	−/−	−/−
	Iron	40%	−/−	−/−	−/−	−/−	−/−	−/+	−/−	−/−	−/−	−/−	+/+	−/+	−/−	−/−	−/−
		60%	−/−	−/−	−/−	−/−	−/−	+/−	−/−	−/−	−/−	−/−	+/+	−/+	−/−	−/−	−/−
		80%	−/−	−/−	−/−	−/−	−/−	+/−	−/−	−/−	−/−	−/−	+/+	−/−	−/−	−/−	−/−

a −/− stands for no viable virus detected for both BA.1 and BA.5; +/− stands for viable virus detected for BA.1 but not BA.5; −/+ stands for viable virus detected for BA.5 but not BA.1; +/+ stands for viable virus detected for both BA.1 and BA.5.

b RH, relative humidity.

**TABLE 3:** Viable virus detected at 37°C^*a*^

Temp	Materials	RH^*b*^	Virus titer														
			1 (Log_10_TCID_50_)					2 (Log_10_TCID_50_)					3 (Log_10_TCID_50_)				
			6h	1d	3d	5d	7d	6h	1d	3d	5d	7d	6h	1d	3d	5d	7d
37°C	Paper carton	40%	−/−	−/−	−/−	−/−	−/−	−/−	−/−	−/−	−/−	−/−	−/−	−/−	−/−	−/−	−/−
		60%	−/−	−/−	−/−	−/−	−/−	−/−	−/−	−/−	−/−	−/−	−/−	−/−	−/−	−/−	−/−
		80%	−/−	−/−	−/−	−/−	−/−	−/−	−/−	−/−	−/−	−/−	−/−	−/−	−/−	−/−	−/−
	Nonwoven fabric	40%	−/−	−/−	−/−	−/−	−/−	−/−	−/−	−/−	−/−	−/−	−/+	−/−	−/−	−/−	−/−
		60%	−/−	−/−	−/−	−/−	−/−	−/−	−/−	−/−	−/−	−/−	−/+	−/−	−/−	−/−	−/−
		80%	−/−	−/−	−/−	−/−	−/−	−/−	−/−	−/−	−/−	−/−	+/+	−/−	−/−	−/−	−/−
	PE packaging film	40%	−/−	−/−	−/−	−/−	−/−	−/−	−/−	−/−	−/−	−/−	−/−	−/−	−/−	−/−	−/−
		60%	−/−	−/−	−/−	−/−	−/−	−/−	−/−	−/−	−/−	−/−	−/+	−/−	−/−	−/−	−/−
		80%	−/−	−/−	−/−	−/−	−/−	−/−	−/−	−/−	−/−	−/−	+/+	−/−	−/−	−/−	−/−
	Iron	40%	−/−	−/−	−/−	−/−	−/−	−/−	−/−	−/−	−/−	−/−	−/−	−/−	−/−	−/−	−/−
		60%	−/−	−/−	−/−	−/−	−/−	−/−	−/−	−/−	−/−	−/−	−/−	−/−	−/−	−/−	−/−
		80%	+/−	−/−	−/−	−/−	−/−	−/−	−/−	−/−	−/−	−/−	+/−	−/−	−/−	−/−	−/−

a −/− stands for no viable virus detected for both BA.1 and BA.5; +/− stands for viable virus detected for BA.1 but not BA.5; −/+ stands for viable virus detected for BA.5 but not BA.1; +/+ stands for viable virus detected for both BA.1 and BA.5.

b RH, relative humidity.

In this study, three different viral titers were applied for inoculation with different materials. With a low virus titer (10 50% tissue culture infectious dose [TCID_50_]), the viable virus of both subvariants was only detected at 6 h, and under very limited conditions, such as BA.1 on the surface of iron at 37°C with an RH of 80% (Table 3), BA.5 on the surface of nonwoven fabric at both 4°C and 25°C with an RH of 80%, on PE packaging film at 4°C with an RH of 40% and 60%, and at 25°C with an RH of 80% (Table 1 and 2). At the middle virus titer (100 TCID_50_), more positive samples were detected, and the time period of viable virus detection extended to 1 day under some conditions, especially at 4°C. With the high virus titer (1,000 TCID_50_), an increased number of samples were positive for the viable virus for prolonged periods of time, and the persistence time of BA.1 and BA.5 on the surfaces of nonwoven fabric, PE packaging film, and iron extended to day 5 and day 7 at 4°C, respectively (Table 1). These results demonstrate that virus viability is titer-dependent. The higher the viral titer, the more likely the virus is to survive.

The virus viability was also varied on different surface types. Among the four tested kinds of materials, both the BA.1 and BA.5 subvariants were more easily inactivated on the surfaces of paper carton, and the survival time of the virus did not exceed 1 day under all conditions (Table 1 and 3). However, the survival time of BA.1 and BA.5 on the surfaces of nonwoven fabric, PE packaging film, and iron could extend to day 5 and day 7, respectively, under certain conditions (high virus titer, 4°C, and an appropriate RH) (Table 1).

RH was also investigated as an environmental factor in this study. With RH values of 40%, 60%, and 80%, a total of 15, 16, and 16 samples were positive for viable BA.1, respectively, while 25, 25, and 18 samples were positive for viable BA.5 at 4°C during the tested period (Table 1). With the increase of temperature to room temperature (25°C), the number of infectious samples decreased to five, eight, and seven using BA.1, and nine, 10, and 10 using BA.5 with RH values of 40%, 60%, and 80%, respectively (Table 2). When the temperature reached 37°C, the viable virus was only found in four samples using BA.1 with an RH of 80%, and one, two, and two samples using BA.5 with RH values of 40%, 60%, and 80%, respectively (Table 3). According to the results, it appears that the BA.5 subvariant is more stable with RH values of 40% and 60% than with an RH value of 80% at 4°C. Further analysis showed that the impact of the RH on the virus stability varied depending on the material type and virus titer. BA.5 could survive for 7 days on nonwoven fabric, PE packaging film, and iron with a high virus titer (1,000 TCID_50_) and RH values of 40% and 60%. Its survival time on the surfaces of these three materials with an RH of 80% was reduced to 5 days (nonwoven fabric and PE packaging film) and 3 days (iron) (Table 1). However, on surfaces treated with a moderate virus titer (100 TCID_50_) and a low virus titer (10 TCID_50_), the trend of an RH of 80% being more detrimental to the virus was only observed on 100 TCID_50_-treated paper carton and iron, and 10 TCID_50_-treated PE packaging film. Taken together, these results suggest that the role of humidity is more intricate, or that it coordinates with other factors.

**FIG 1:**
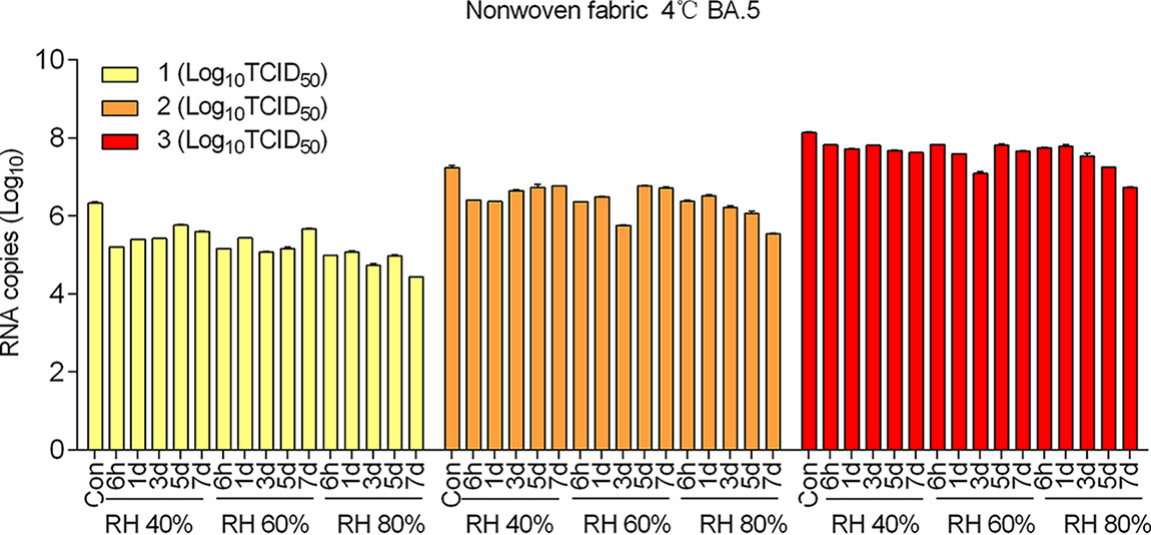
Virus nucleic acid copies of samples recovered from Omicron BA.5-inoculated nonwoven fabric at 4°C. A total of 400 μL of recovered sample was used for nucleic acid extraction, and the viral RNA copy numbers were measured via reverse transcription-PCR (RT-PCR) using primers and a probe targeting the SARS-CoV-2 N gene. Quantification was calculated according to the standard curve. The results are expressed as the mean ± SD. RH, relative humidity.

**FIG 2:**
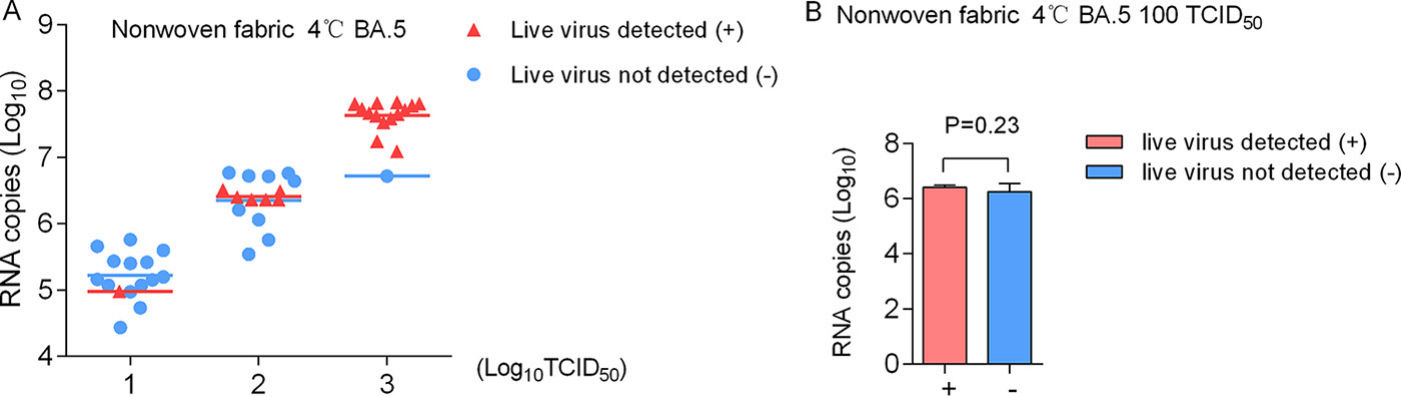
Virus nucleic acid copy numbers of samples with live virus detected (+) and not detected (−) from Omicron BA.5-inoculated nonwoven fabric at 4°C. (A) Scatter diagram of RNA copy numbers from samples inoculated with different virus titers. Samples detected with or without live virus are differentially displayed. (B) RNA copy numbers of samples with live virus detected (+) and not detected (−) from 100 TCID_50_ BA.5-inoculated nonwoven fabric at 4°C. The results are expressed as the mean ± SD. The values were evaluated with Student's *t* test.

This work further explored the relationship between virus nucleic acid copy numbers and virus viability by using samples of Omicron BA.5-inoculated nonwoven fabric at 4°C, as they exemplified the group with the highest positive rate. The virus nucleic acid results were completely different from the results of virus viability, and showed high stability on surfaces of nonwoven fabric over the whole study period (Fig. 1). When the virus nucleic acid copy numbers of samples with or without live virus detected in this group (Omicron BA.5-inoculated nonwoven fabric at 4°C) were represented on a scatter diagram, no significant separations were observed (Fig. 2A). Then, the virus nucleic acid copy numbers between samples with and without live virus detected were statistically analyzed in one subgroup (100 TCID_50_ BA.5-inoculated nonwoven fabric at 4°C). The results showed that no discrepancy existed (*P = *0.23) (Fig. 2B), suggesting that virus nucleic acid is relatively stable on materials regardless of whether viable virus can be detected. These findings indicate that the detection of virus nucleic acid on surfaces does not directly indicate the existence of infectious virus.

## DISCUSSION

Contaminated fomites are thought to be a potential transmission route in the current pandemic caused by SARS-CoV-2 (3, 4). These contact transmission events are more likely to happen in the express and logistics industry due to the high volume of transported cargos. Once transport items or their packages are contaminated, onward transmission has the potential to occur.

This study evaluated the environmental stability of two Omicron subvariants on the surfaces of four widely used transport packaging materials. Among these four materials, the Omicron variant was rapidly inactivated on the surface of paper carton, while it persisted for a longer period of time on the surfaces of nonwoven fabric, PE packaging film, and iron. These results are consistent with previous reports that SARS-CoV-2 has a long survival time on nonporous material or porous material made up of high-density hydrophobic ingredients, such as the outer layer of surgical masks and Tyvek (3, 5, 6). Compared with the other three materials used in this study, paper carton has good water absorptivity, which is also an important reason why viruses are more easily inactivated on paper carton. On hydrophobic materials, the virus was more stable as the viral particles inside droplets may have remained infective for a longer time. However, on material surfaces with good water absorptivity, the virus droplets were absorbed and the viral particles were easily inactivated (3).

The environmental stability of SARS-CoV-2 has been compared with other viruses and between different SARS-CoV-2 VOCs (2, 7–11). These previous studies demonstrated that the viral survival time varied between different strains. SARS-CoV-2 VOCs, especially Omicron variants, have higher environmental stability than the original strain (2). However, the same degree of environmental stability has been demonstrated between SARS-CoV-2 Alpha and Beta variants, and between Omicron BA.1 and BA.2 (2, 11). To date, there have been no reports describing the stability of Omicron BA.1 and BA.5. In the current study, Omicron BA.1 and BA.5 were used as the research strains, and Omicron BA.5 exhibited a higher degree of environmental stability than BA.1. These data provide valuable information that can be used as a reference by other research groups. In addition, more in-depth research is needed to explore the environmental stability of new variants and investigate the potential mechanisms involved.

In this study, the effects of the temperature and RH on the stability of SARS-CoV-2 Omicron variants were investigated. The stability of viruses under different temperatures was consistent with previous studies that indicated that increases in temperature accelerated the inactivation of SARS-CoV-2 on surfaces (12, 13). However, the present study did not observe a definite effect of RH on viral stability. The effect of RH may be much more complicated, whereas other factors, such as the water absorptivity of the materials and viral titers, should be considered intensively. The relationship between viral inactivation and RH was complicated, as reported in a previous study (12). Future studies should be performed intensively to understand the effect of humidity on virus stability.

Of note, while viable viruses decayed over an extended time, the virus nucleic acid showed high stability on material surfaces. In real-life settings, reverse transcription-PCR (RT-PCR) is widely used to determine the contamination of the virus in environment samples. However, it should be noted that a positive result of virus nucleic acid, even with low cycle threshold (CT) values, does not mean that infectious viruses exist. The results of RT-PCR should be interpreted and evaluated cautiously.

In conclusion, this study demonstrates that the environmental stability of the SARS-CoV-2 Omicron variant varies based on surface type. The virus viability on surfaces was highly dependent on both the temperature and viral titer. Low temperatures and high viral titers promote the persistence of the virus. Moreover, in contrast to virus viability, virus nucleic acid has high stability on widely used materials, and is not suitable for evaluating the risk of epidemic transmission.

## MATERIALS AND METHODS

### Viruses and cells.

The SARS-CoV-2 variants analyzed in this study were the Omicron BA.1 and BA.5 strains. The BA.1 virus (IPBCAMS-OM01-1/2021) was isolated from a clinical sample; BA.5 (CCPM-B-V-049-2207-28) was provided by the Institute of Laboratory Animal Sciences, Chinese Academy of Medical Sciences & Peking Union Medical College. Vero cells (African green monkey kidney cells) were purchased from American type culture collection (ATCC) (CCL-81), cultured in Dulbecco’s modified Eagle Medium (DMEM, Gibco) supplemented with 10% fetal bovine serum (FBS), and used for virus culture and qualification. All work handling SARS-CoV-2 was performed in a Biosafety Level 3 (BSL-3) laboratory.

### Preparation of test materials.

The materials used in this study were widely used transport packaging materials provided by Beijing Customs, including paper cartons, nonwoven bags, PE packaging film, and iron drum packaging. All materials were cut into small pieces of about 1.5 cm^2^. PE packaging film was sterilized using 75% alcohol immersion. Other materials were sterilized via autoclaving. Pieces of materials were then placed in 12-well plates for inoculation with viral aliquots.

### Inoculation with test surfaces.

The titers of Omicron BA.1 and Omicron BA.5 virus stocks were determined based on the 50% TCID_50_ according to our previous report (14). The virus stocks were thawed immediately before use. Then, 20-μL droplets of viral suspension containing 10 TCID_50_, 100 TCID_50_, and 1,000 TCID_50_ virus were deposited onto the surface of the pre-prepared materials in 12-well plates. The plates were placed into sealed boxes with different levels of relative humidity (40%, 60%, and 80%) and incubated under various temperatures (4°C, 25°C, and 37°C).

### Viable virus detection.

The virus was recovered from the surfaces of the materials at each time point by adding 2.0 mL DMEM to each sample. Then, 200 μL of DMEM collected from each sample was used for incubation with Vero cells. The cytopathic effects (CPEs) were observed 4 days later. If no CPEs were observed, the supernatants were collected and passaged three times. The observed CPEs in any generation were recorded to indicate the activities of the virus.

### Virus nucleic acid quantification.

Four hundred μL of each recovered sample was used for nucleic acid extraction with a Direct-zol RNA miniPrep kit (Zymo Research, Irvine, CA, USA) according to the manufacturer’s instructions. The virus nucleic acid copy numbers were measured using RT-PCR with primers and a probe targeting the SARS-CoV-2 N gene. Quantification was performed using the N target with a standard curve generated based on the serial dilution of the reference standard.
